# Favourable clinical outcomes and low revision rate after M‐ACI in adolescents with immature cartilage compared to adult controls: Results at 10 years

**DOI:** 10.1002/ksa.12359

**Published:** 2024-07-15

**Authors:** Johannes Weishorn, Johanna Wiegand, Kevin‐Arno Koch, Raphael Trefzer, Tobias Renkawitz, Tilman Walker, Yannic Bangert

**Affiliations:** ^1^ Department of Orthopaedics Heidelberg University Hospital, Ruprecht‐Karls‐University Heidelberg Heidelberg Germany

**Keywords:** adolescent, cartilage, clinical study, knee, magnetic resonance imaging

## Abstract

**Purpose:**

The purpose of this study was to evaluate long‐term survival, patient‐reported outcomes (PROs) and radiographic results of matrix‐associated autologous chondrocyte implantation (M‐ACI) in adolescents with immature cartilage and compare them to adult controls.

**Methods:**

A retrospective matched‐pair analysis was performed comparing the PRO after M‐ACI for focal cartilage defect of the knee in cartilaginous immature adolescents to mature adults. Groups were matched for sex, body mass index, defect site and size, symptom duration and the number of previous knee surgeries. Knee Injury and Osteoarthritis Outcome Score (KOOS) and the Magnetic Resonance Observation of Cartilage Repair Tissue (MOCART 2.0) scores were assessed at least 60 months postoperatively. Patient acceptable symptomatic state (PASS) and clinical response rate in KOOS and KOOS subscores were calculated.

**Results:**

A total of 54 patients were matched. At a mean of 96 months (65–144 months), no surgical complications, graft hypertrophy or reoperations were noted in the cohorts studied. Adolescents showed superior PROs at the final follow‐up (76.9 ± 14.1 vs. 66.4 ± 15.0, *p* = 0.03) and were significantly more likely to achieve PASS (74.1% vs. 55.6%; *p* = 0.02) compared to the adult cohort. The KOOS subscale analysis showed long‐term benefits for adolescents in terms of symptom improvement, pain reduction, activities of daily living, sports and quality of life (*p* < 0.05). None of the patients in the adolescent group showed graft hypertrophy on magnet resonance imaging or signs of osteoarthritis on radiographs at long‐term follow‐ups.

**Conclusions:**

M‐ACI is an effective treatment for chondral defects of the knee in patients with immature cartilage with low revision rates and high patient satisfaction over the long term. Adolescents showed comparable clinical and radiographic results in the short and medium term, with slightly more favourable, clinically relevant functional results in adolescents in the long term. M‐ACI can be safely used in adolescents, and consideration should be given to expanding the indication to include these patients.

**Level of Evidence:**

Level III.

**Abbreviations**
ADL: activities of daily living
AMADEUS: area measurement and depth and underlying structures
BMI: body mass index
CI: confidence interval
CRR: clinical response rate
DFO: distal femoral osteotomy
DGOU: German Society for Orthopedics and Trauma Surgery
EQ5D: European Quality of Life 5 Dimensions
FU: follow‐up
HTO: high tibial osteotomy
ICRS: International Cartilage Repair Society
KOOS: Knee Injury and Osteoarthritis Outcome Score
MCID: minimal clinically important difference
MOCART: Magnetic Resonance Observation of Cartilage Repair Tissue
MRI: magnet resonance imaging
OA: oteoarthritis
OCD: osteochondritis dissecans
PASS: patient acceptable symptomatic state
PRO(M): patient‐reported outcome (measures)
PSM: propensity score matching
QOL: quality of life
SD: standard deviation
T: Tesla
VAS: Visual Analogue Scale
ΔKOOS: difference in KOOS from preoperative values
(M‐)ACI: matrix‐associated autologous chondrocyte implantation

## INTRODUCTION

Articular cartilage defects predispose to the early onset of osteoarthritis (OA) and are, therefore, serious pathologies, especially in young and active patients [9]. There are several techniques used to repair focal articular defects in adolescents. Bone marrow stimulating (BMS) procedures such as microfracture or microdrilling have shown satisfactory regenerative potential in young patients [2, 21]. However, there are serious concerns about the long‐term performance of the repair tissue [23]. Cartilage regenerative techniques such as osteochondral allografts, osteochondral transplants and matrix‐associated BMS are either rare or limited to medium‐sized defects [20]. Matrix‐associated autologous chondrocyte implantation (M‐ACI) has shown promising long‐term functional results and low mid‐ to long‐term failure rates in adults, but its use in skeletally immature patients is limited by regulatory barriers [8, 20, 32]. From a scientific perspective, the age restriction of M‐ACI in adolescent patients is difficult to understand. Niemeyer et al. showed that patients aged less than 20 years had a higher expression rate of cartilage‐specific markers on chondrocytes and thus a higher chondrocyte quality after culturing in vitro [22, 27]. Thus, because their articular cartilage is still in the process of maturation, adolescents under the age of 20 years may have a greater chondrogenic potential of chondrocytes in M‐ACI [22, 27].

According to a recently published registry study, failed cartilage repair is associated with a worse clinical outcome in revision M‐ACI, regardless of the type of previous cartilage repair [29]. Despite a worse clinical outcome and alarming rates of cartilage degradation at 5 years, alternative procedures such as BMS techniques must be used in these patients [11, 20]. However, the use of M‐ACI in immature adolescents is difficult to justify due to limited clinical evidence.

Currently, there are few studies evaluating the outcome of ACI in adolescent patients [13, 16, 17, 18, 24, 26]. These studies report a concerning rate of graft failure with first‐generation ACI in adolescents, with 20%–69% requiring surgical revision [6, 16, 26]. The primary reason for revision was graft hypertrophy [26]. For third‐generation M‐ACI, the revision rate has been reported to be between 3% and 10% [13, 24]. However, the relevance of these studies is limited by short follow‐up and an unmatched comparison group without adjustment for potential confounders [13, 24].

This study aims to address this gap by evaluating the long‐term survival, clinical and radiographic outcomes of M‐ACI in adolescents and comparing them to a propensity score‐matched adult cohort. It was hypothesised that M‐ACI in adolescents with immature cartilage would have similar long‐term clinical and radiographic outcomes with low rates of graft failure compared to adult controls.

## MATERIALS AND METHODS

### Study design

Data on patients who underwent cartilage repair for focal cartilage damage of the knee joint were collected in an institutional review board‐approved database at the single‐centre academic institution where the present study was conducted. The present study was approved by the Institutional Review Board of the University of Heidelberg (S‐029/021) and was conducted in accordance with the 2008 Declaration of Helsinki. Board‐certified orthopaedic surgeons were responsible for enroling participants into the database.

**Figure 1:**
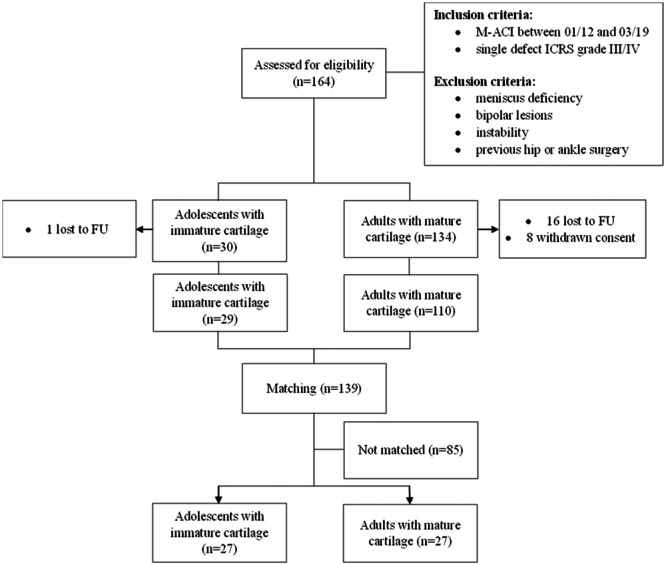
Flowchart visualising patient selection and matching. BMI, body mass index; FU, follow‐up; M‐ACI, matrix‐associated autologous chondrocyte implantation; PSM, propensity score matching.

This retrospective cohort study enroled 164 patients from the above database based on defined inclusion and exclusion criteria. Patients who underwent M‐ACI for a single International Cartilage Repair Society III or IV cartilage defect at the knee between January 2012 and March 2019 were included in the study. Patients with more than one‐third meniscal deficit, history of ligamentous instability, bipolar lesions or previous hip or ankle surgery were excluded (Figure 1).

Based on the inclusion and exclusion criteria, 30 patients younger than 20 years with immature articular cartilage were identified, of whom 29 (97%) were available for long‐term follow‐up. Subsequently, database‐driven propensity score matching (PSM) was performed for sex, body mass index (BMI), lesion size and location and number of previous knee surgeries to obtain two cohorts with comparable demographic characteristics.

The PSM resulted in two cohorts with homogeneous baseline characteristics (Table 1).

**Table 1:** Baseline demographic characteristics of M‐ACI‐treated adolescents and adults (*n* = 54).

Characteristics	Adolescents				Adults				*p* Value
No. of patients	27				27				
Follow‐up (years)	8.0 (5.4–11.8)				8.3 (5.7–12.0)				(n.s.)
Age (years)	17.5 (13.3–19.1)				32.4 (20.6–44.2)				<0.001
Sex (%)									
Male	13				13				(n.s.)
Female	10				10				
BMI (kg/m²)	25.2 (3.5)				25.1 (4.1)				(n.s.)
Symptom duration (mo.)	10.4 (12.1)				13.5 (12.9)				(n.s.)
Defect size (cm²)	4.6 (2.0)				4.6 (1.8)				(n.s.)
ICRS grade	**III**		**IV**		**III**		**IV**		(n.s.)
	3		24		3		24		
Localisation (%)	**FT**		**PF**		**FT**		**PF**		(n.s.)
	19		8		19		8		
Previous knee surgeries	**0**	**1**	**2**	**≥3**	**0**	**1**	**2**	**≥3**	(n.s.)
	18	7	2	0	18	8	1	0	

*Note*: Bold values are used for categorical differentiation of patient characteristics.

Abbreviations: BMI, body mass index; FT, femorotibial; ICRS, International Cartilage Repair Society; M‐ACI, matrix‐associated autologous chondrocyte implantation, PF, patellofemoral.

### Surgical procedure and rehabilitation

Adolescent patients with open or incompletely closed growth plates were educated about the off‐label use of M‐ACI. The indication for M‐ACI was confirmed by diagnostic arthroscopy followed by chondrocyte harvest. After 4 weeks, implantation of chondrocytes cultured on a type I/III collagen matrix (Novocart 3D®, TETEC) was performed through a mini‐open arthrotomy. First, the defect site was prepared by creating a stable marginal wall of vital articular cartilage. Subchondral sclerosis was removed, and in cases of subchondral bone involvement >2 mm, additional autologous cancellous bone grafting was performed. The chondrocyte‐seeded membrane was fixed with resorbable sutures and fibrin glue.

After an initial immobilisation period of 48 h, range of motion was released according to the location of the defect, and a structured, physiotherapist‐supervised exercise programme was initiated. Patients were instructed to use a continuous passive motion device several times a day for the first 6 weeks. Full range of motion was allowed at 6 weeks postoperatively. Sport‐specific loading was allowed 12 months postoperatively. Postoperative management was individualised with respect to defect location, degree of graft healing, concomitant procedures and patient activity level.

Concomitant surgery was performed as indicated. Comorbidities such as tibiofemoral malalignment >3° or lateralized patellar tracking were corrected with tibial and femoral osteotomies. Tibiofemoral malalignment was corrected with distal femoral osteotomies and high tibial osteotomies and fixed with a locking plate (TomoFix®, DePuy Synthes). Preoperative and postoperative leg alignment was categorised using limb phenotypes as described by Hirschmann et al. [12, 14]. With concomitant osteotomies, straight leg alignment was achieved in the majority of patients (Table 2).

**Table 2:** Preoperative and postoperative alignment information of the patients treated.

	Adolescents (*n*)		Adults (*n*)	
HKA	Preoperative	Postoperative	Preoperative	Postoperative
VAR9	2		1	
VAR6	2		3	
VAR3	2		1	1
Neutral	19	25	19	25
VAL3	1	2	3	1
VAL6	1			
VAL9				

Abbreviations: HKA, hip–knee angle; VAR, varus; VAL, valgus.

Lateralized patellar tracking was corrected with an tibial tubercle osteotomy and additional soft tissue balancing (e.g., z‐shaped lateral retinaculum lengthening). The tibial tubercle osteotomy was fixed with two full‐threaded steel screws after the required correction. The patient was advised to take vitamin D3 supplements and not to smoke.

A total of 12 adolescent patients had open growth plates at the time of surgery, five were partially open and 10 were closed. One or more concomitant procedures were performed in 15 of the adolescents and 14 of the adults (Table 3). Osteochondritis dissecans (OCD) was the primary reason for M‐ACI in adolescents, whereas OCD and trauma were the predominant etiologies in adults.

**Table 3:** Patient‐ and procedure‐specific baseline characteristics.

Characteristics	Adolescents	Adults
Physeal status (*n*)		
Open	12	0
Partially open	5	0
Closed	10	27
Concomitant procedures (*n*)		
TTO + DFO	1	0
DFO	3	2
HTO	4	4
Autologous bone grafting	12	8
No concomitant procedure	12	13
Lesion type (*n*)		
Traumatic	11	10
OCD	16	10
Degenerative	0	7
Defect location (*n*)		
Medial femoral condyle	12	13
Lateral femoral condyle	7	6
Patella	7	7
Trochlea	1	1

Abbreviations: DFO, distal femoral osteotomy; HTO, high tibial osteotomy; OCD, osteochondritis dissecans; TTO, tibial tubercle osteotomy.

### Outcome measures

Clinical results were assessed using patient‐reported outcome questionnaires at the time of surgery and at 12, 24 and at least 60 months postoperatively to determine the Knee Injury and Osteoarthritis Outcome Score (KOOS) and the difference in KOOS from preoperative values (ΔKOOS) [19]. The mean time to follow‐up for the long‐term evaluation was 96 months. Additional patient‐reported outcome (measures) to assess pain level (visual analogue scale [VAS]), function (IKDC), quality of life (European Quality of Life 5 Dimensions) and activity level (Tegner) were also recorded and are listed in the appendix (Table S1). Treatment failure was defined as the need for reoperation for any reason.

The previously published patient acceptable symptomatic state (PASS) values were used for precise assessment and categorisation of the KOOS [4]. The minimal clinically important difference (MCID) of the KOOS was set at 10 in accordance with previous literature and recommendations [25]. The clinical response rate (CRR), defined as the percentage of patients achieving the MCID, was also calculated [33].

Analysis of magnetic resonance imaging (MRI) scans was performed preoperatively and postoperatively on a 3 T high‐resolution MRI. Preoperatively and 12 and 24 months postoperatively, scans were performed on a 3 T scanner (Magnetom Verio; Siemens Healthineers) with an 18‐channel total imaging matrix in combination with a dedicated 15‐channel knee coil. Due to infrastructure changes at the academic institution, a new generation 3 T MR scanner (Magnetom Vida; Siemens Healthineers) with an 18‐channel knee coil was used for the long‐term follow‐up evaluation. The imaging sequences were equivalent for both scanners and included high‐resolution proton density‐weighted turbo spin‐echo (with and without fat saturation) in all imaging planes and T1 spin‐echo sequences in the coronal and sagittal planes. MRI assessment was performed preoperatively with the area measurement and depth and underlying structures score and postoperatively with the Magnetic Resonance Observation of Cartilage Repair Tissue (MOCART) 2.0 score by two blinded orthopaedic surgeons.

All patients underwent preoperative leg alignment evaluation. Patients who underwent an additional osteotomy were reassessed postoperatively. OA was evaluated preoperatively and at least 60 months postoperatively using radiographs of the knee in two planes and graded I–IV.

### Statistical analysis

To reduce bias from potential confounders, a 1:1 nearest neighbour PSM with replacement was performed. The tolerance for matching was set at 0.01 to obtain groups with similar baseline characteristics. Priority was given to exact matching without minimising memory and with shuffling enabled. The PSM resulted in two groups of 27 subjects each with similar baseline characteristics.

Clinical and radiographic data were described as absolute and relative values or as means with standard deviation or range. The *χ* ^2^ test was used to compare categorical variables between two groups. Continuous variables were compared by unpaired *t* test or Wilcoxon rank‐sum test in case of nonparametric dispersion. The significance level was set at 0.05. Bonferroni correction was not required.

Intraclass correlation coefficients showed excellentintraobserver (0.94 [95% confidence interval, CI 0.89–0.97]) and good interobserver (0.86 [95% CI 0.70–0.94]) reliability of the radiographic assessment. Statistics and matching were performed using SPSS software version 29.0 (IBM) and G‐Power 3.1 (Heine University, Düsseldorf, Germany).

A post hoc power analysis was performed to interpret the results. When analysing clinical data at 96 months postoperatively, a calculated effect size of *ω* = 0.769 with an available patient number of *n* = 27 and an *α* set at 0.05 provides a calculated statistical power of 79.2% to detect an underlying difference in KOOS.

## RESULTS

### Complications and reoperation rate

No surgical complications were observed in the study cohort. None of the patients who underwent M‐ACI required revision at the current follow‐up. In the adolescent group, 93% of patients would choose to undergo surgery again, compared to 70% in the adult group (*p* = 0.03).

### Clinical analysis

Adolescents achieved significantly higher KOOS (76.9 ± 14.1 vs. 66.1 ± 14.0; *p* = 0.03) and were more likely to achieve PASS (*p* = 0.02) at long‐term follow‐ups (Table 4).

**Table 4:** Comparison of the KOOS score and PASS at the various FUs of the matched cohort.

	Adolescents	Adults	*p* Value
Baseline	48.4 (30.1)	52.0 (27.0)	(n.s.)
12 Months	54.3 (19.7)	57.4 (23.1)	(n.s.)
% reach PASS	29.6	33.3	(n.s.)
24 Months	68.9 (14.9)	63.0 (16.7)	(n.s.)
% reach PASS	51.9	44.4	(n.s.)
96 Months	76.9 (14.1)	66.1 (14.0)	0.03^a^
% reach PASS	74.1	55.6	0.02^a^

*Note*: Mean (SD).

Abbreviations: FU, follow‐up; KOOS, Knee Injury and Osteoarthritis Outcome Score; PASS, patient acceptable symptomatic state.

^a^ Significance.

Differences in ΔKOOS between the two groups were significant at long‐term follow‐ups (*p* = 0.02; Table 5). Both groups showed acceptable CRRs at 96 months (69.6% vs. 60.9%; *p* = n.s.).

**Table 5:** Comparison of ΔKOOS score from baseline to the different FUs and CRR at various FUs in the matched cohort.

	Adolescents	Adults	*p* Value
12 Months	5.9 (36.4)	5.4 (33.9)	(n.s.)
CRR in %	55.6	48.1	(n.s.)
24 Months	20.5 (34.5)	12.0 (31.1)	0.03
CRR in %	66.7	51.9	0.02^a^
96 Months	28.4 (36.9)	16.3 (31.3)	0.02^a^
CRR in %	70.4	66.7	(n.s.)

*Note*: Mean (SD).

Abbreviations: CRR, clinical response rate; FU, follow‐up; KOOS, Knee Injury and Osteoarthritis Outcome Score.

^a^ Significance.

The KOOS subscale analysis showed long‐term benefits for adolescents in terms of symptom improvement (60.1 ± 12.4 vs. 53.7 ± 14.0), pain reduction (86.2 ± 15.8 vs. 80.4 ± 15.9), activities of daily living (93.9 ± 9.3 vs. 85.2 ± 15.8), sports (72.5 ± 18.1 vs. 61.7 ± 15.6) and quality of life (71.1 ± 22.3 vs. 60.7 ± 16.1) (Table 6).

**Table 6:** KOOS subcomponent analysis indicating significant differences between both groups (*p* ≤ 0.05).

	Symptoms	Pain	ADL	Sports	QOL
Baseline	(n.s.)	(n.s.)	(n.s.)	(n.s.)	(n.s.)
12 Months	0.02^a^	0.01^a^	(n.s.)	(n.s.)	(n.s.)
24 Months	(n.s.)	0.02^a^	(n.s.)	0.02^a^	(n.s.)
96 Months	0.01^a^	0.01^a^	0.01^a^	0.01^a^	0.01^a^

Abbreviations: ADL, activities of daily living; KOOS, Knee Injury and Osteoarthritis Outcome Score; QOL, quality of life.

^a^ *p* Values, significance.

**Figure 2:**
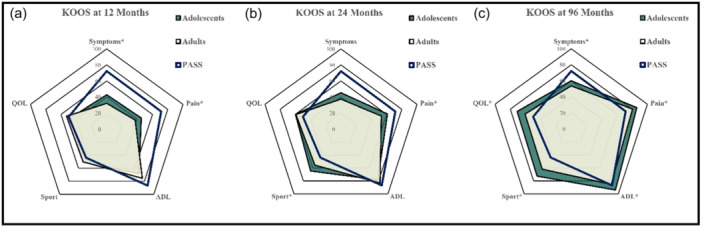
Differences in Knee Injury and Osteoarthritis Outcome Score (KOOS) subscores between the two groups at 12 (a), 24 (b) and 96 months (c) in relation to patient acceptable symptomatic state; ADL, activities of daily living; PASSpatient acceptable symptomatic state; QOL, quality of life. *Significance.

The differences in KOOS subscores at 12, 24 and 96 months are shown in Figure 2 in relation to the PASS.

**Figure 3:**
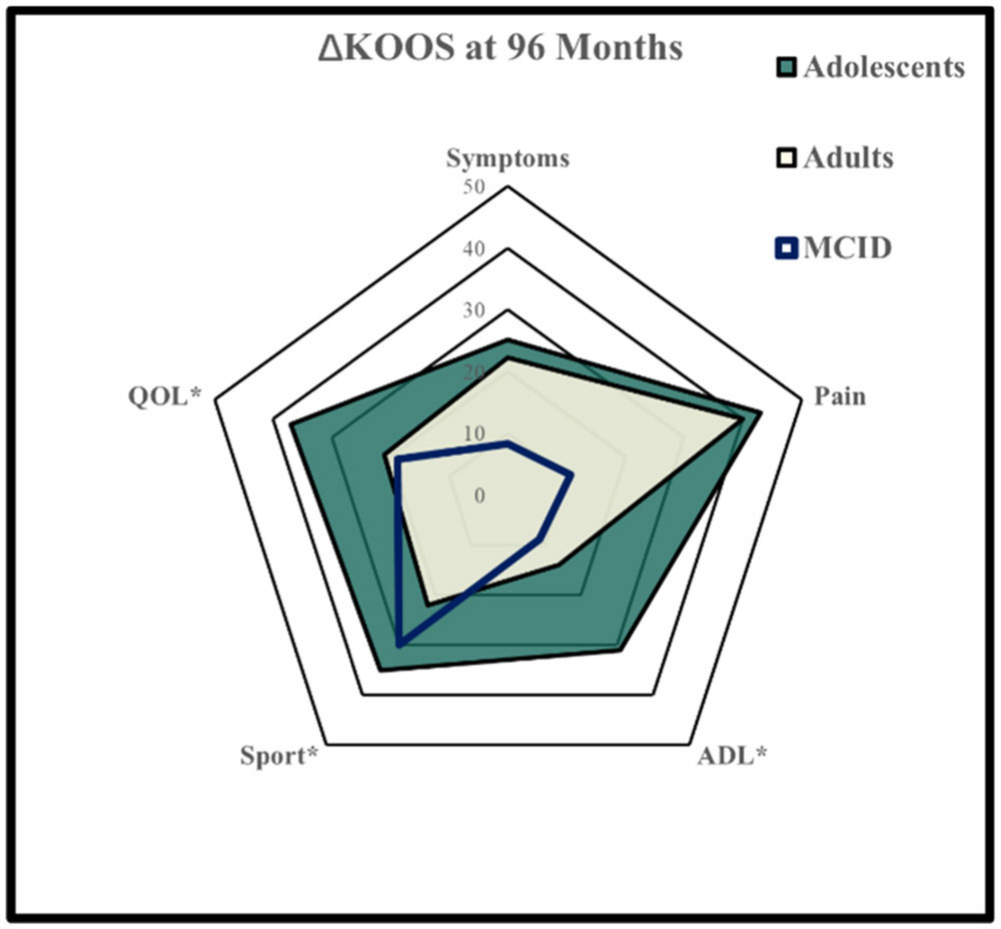
Differences in Knee Injury and Osteoarthritis Outcome Score (KOOS) subscores between the two groups at 96 months in relation to minimal clinically important difference (MCID). ADL, activities of daily living; QOL, quality of life; MCID, minimal clinically important difference. *Significance.

The MCID for the KOOS subscores at long‐term follow‐ups was largely met in both groups. However, adults did not meet the MCID for KOOS Sport. Significant benefits for adolescents were seen in the ΔKOOS subscales for sport, quality of life and activities of daily living (*p* < 0.05; Figure 3).

Stratified by limb alignment, patients with a postoperative valgus phenotype had a slightly inferior KOOS at various follow‐ups compared to patients with a straight or slightly varus phenotype (Table 7).

**Table 7:** KOOS among the postoperative limb phenotypes at various FUs.

	Adolescents				Adults			
KOOS	*n*	12 Months	24 Months	96 Months	*n*	12 Months	24 Months	96 Months
VAR3					1	65.8	71.0	78.4
Neutral	25	54.5 (19.9)	69.3 (15.1)	77.3 (14.2)	25	57.1 (24.9)	62.9 (18.1)	66.0 (14.5)
VAL3	2	52.1 (9.5)	64.4 (11.2)	72.1 (13.0)	1	56.0	58.4	55.2

Abbreviations: FU, follow‐up; KOOS, Knee Injury and Osteoarthritis Outcome Score; VAL, valgus; VAR, varus.

### Radiographic analysis

Preoperative and postoperative MRI scans were available for radiologic evaluation in all 54 matched patients at long‐term follow‐ups (Table 8). Adolescent patients showed consistently high MOCART scores at 24 and 96 months, respectively (83.2 ± 11.5 and 80.3 ± 16.3; *p* = n.s.). None of the patients in the adolescent group showed graft hypertrophy on MRI or signs of OA on radiographs. In the adult control group, one patient showed progression of OA from preoperative Kellgren–Lawrence grades I–III at 7.6 years postoperatively. Three patients had preoperative grade I OA without progression during follow‐up.

**Table 8:** Comparison of the AMADEUS/MOCART scores at the various FUs of the matched cohort.

	Adolescents	Adults	*p* Value
AMADEUS	30.4 (15.7)	39.0 (14.6)	(n.s.)
MOCART			
12 Months	76.1 (16.8)	84.2 (13.4)	(n.s.)
24 Months	78.4 (18.3)	80.0 (18.2)	(n.s.)
96 Months	80.6 (16.6)	76.2 (19.2)	(n.s.)

*Note*: Mean (SD).

Abbreviations: AMADEUS, area measurement and depth and underlying structures; FU, follow‐up; MOCART, Magnetic Resonance Observation of Cartilage Repair Tissue.

## DISCUSSION

The main finding of the study is that M‐ACI in an adolescent population is associated with low revision rates comparable to those in adults. Adolescent patients achieve PASS more frequently (74.1% vs. 55.6%; *p* = 0.02) and with a clinically relevant superior KOOS (76.9 ± 14.1 vs. 66.4 ± 15.0, *p* = 0.03) at 96 months. Radiographic evaluation demonstrated that none of the patients in the adolescent group showed graft hypertrophy on MRI or signs of OA on radiographs at long‐term follow‐ups.

M‐ACI is a well‐established treatment for focal chondral and osteochondral cartilage lesions of the knee [5, 7, 33]. However, in the adolescent population, periosteal flap ACI has shown concerning rates of graft failure and revision surgery [6, 16, 26]. The incidence of graft hypertrophy ranged from 9% to 40% and was mostly treated with arthroscopic chondral debridement [1, 26]. However, this technique of cartilage regeneration is outdated, but there are currently insufficient data for the treatment of M‐ACI in adolescents [30]. Niethammer et al. report a revision rate of 10% at 36 months after M‐ACI, with a rate of 7.5% (three patients) for graft‐related complications [24]. The partial graft failure due to insufficient graft integration in these three cases was treated with arthroscopic microfracture in a second procedure. In a second study by Hoburg et al., graft‐related treatment failure was 3%, with only one revision for graft hypertrophy [13]. This is essentially consistent with the results of the present study. After an average of 96 months, none of the adolescent patients required revision, and no cases of graft hypertrophy occurred. These results encourage orthopaedic surgeons to perform M‐ACI in patients with immature articular cartilage when indicated to avoid treatment failure due to less reliable alternative cartilage repair [29, 31].

The results of the present study regarding long‐term clinical and radiographic outcomes after M‐ACI in adolescents are promising. The clinical outcome was comparable to previously published data where Hoburg et al. reported an overall KOOS of 82.6 at a mean of 63 months in adolescents treated with M‐ACI [13]. The present study showed comparable short‐ to medium‐term KOOS in both groups, while long‐term results were superior in the adolescent group. Interestingly, Hoburg et al. found no significant differences in outcome compared to the adult population when analysing the KOOS including its subscores [13]. This may be due to the lack of matching of the comparison group in this study and therefore the confounding of the clinical scores by other potential factors influencing clinical outcome such as BMI, number of previous surgeries or gender as recently published [3, 10, 13]. There has been much debate and conflicting results regarding the effect of age on clinical outcomes after ACI. While some studies have shown favourable outcomes in younger patients, others have found no correlation with age [7, 15, 17, 34]. However, different age cut‐offs make it difficult to directly compare these studies. Niethammer et al. were able to demonstrate a clinical advantage for adolescent patients younger than 20 years in terms of IKDC score and VAS score [24]. As in the present study, this age cut‐off is based on the findings of Niemeyer et al., who suggested an age of 20 years as a cut‐off for cartilage maturity between adults and adolescents [22, 27, 28]. Closure of the epiphyseal growth plates precedes and appears to be independent of the process of articular cartilage maturation [28].

Hoburg et al. also performed radiographic assessment, whereas Niethammer et al. only reported clinical outcomes after M‐ACI in adolescent patients. They found a mean MOCART score of 75 for the adolescent patients and 77 for the adult patients in the mid‐term interval after an average of 63 months, with the short‐term interval not reported [13]. The present study found a comparable MOCART score in both groups at long‐term follow‐up. Notably, despite high functional demands, the adolescent group showed excellent radiographic results with good graft maturation and integration without long‐term OA progression.

In conclusion, this is one of the first studies to compare M‐ACI in adolescents and adults. Adolescents showed comparable clinical and radiographic outcomes in the short and medium term, with slightly more favourable, clinically relevant functional outcomes in adolescents in the long term. M‐ACI can be safely used in adolescents, and consideration should be given to expanding the indication to include these patients.

This study has several limitations that need to be discussed. When interpreting the results of the present study, it is important to consider the small number of patients in the cohorts. Although the power calculation shows an acceptable value of 79%, there is still a certain risk of type II error. Therefore, the results should be interpreted with caution. Multicentre studies with larger cohorts may provide further evidence for young patients in the future. In addition, several concomitant surgical procedures, such as alignment correction and autologous bone grafting, were performed in some of the patients studied. This is essential to provide an optimised biomechanical and local osseous environment for M‐ACI grafts and reflects the reality of care in daily practice. Nevertheless, concomitant procedures may confound the clinical and radiographic results of the study.

## CONCLUSION

M‐ACI is an effective treatment for chondral defects of the knee in patients with immature cartilage with low revision rates and high patient satisfaction at 96 months. Adolescents showed comparable clinical and radiographic results in the short and medium term, with slightly more favourable, clinically relevant functional results in adolescents in the long term. M‐ACI can be safely used in adolescents, and consideration should be given to expanding the indication to include these patients. This may avoid failures due to less reliable cartilage regeneration techniques.

## AUTHOR CONTRIBUTIONS

Johannes Weishorn supervised the study and performed data extraction, statistical analysis and drafted the manuscript. Johanna Wiegand performed data collection and were involved in reviewing the manuscript. Kevin‐Arno Koch, Raphael Trefzer and Tilman Walker were involved in reviewing and drafting the manuscript. Tobias Renkawitz critically reviewed and revised the manuscript. Yannic Bangert was involved in interpreting the statistical results and drafting the manuscript.

## CONFLICTS OF INTEREST STATEMENT

Renkawitz Tobias declares the following conflicts of interest: Financial interests: Research funding at personal disposal: DePuy, Zimmer, Aesculap, German Federal Ministry of Education and Research, Deutsche Arthrose‐Hilfe, OttoBock‐Stiftung, German Federal Ministry of Economic and Development, Oskar‐Helene‐Heim Foundation in Berlin, Vielberth Foundation, Deutsche Forschungsgemeinschaft (DFG). Reimbursement of costs: DePuy, Zimmer, Aesculap, Federal Ministry of Education and Research, Deutsche Arthrose‐Hilfe, OttoBock‐Stiftung, Federal Ministry for Economic Co‐operation and Cooperation and Development, Oskar‐Helene‐Heim Foundation in Berlin, Vielberth Foundation, DGOOC, BVOU, DGOU.—Reimbursement of costs for training/lectures: DePuy, Zimmer, Aesculap, German Society for Endoprosthetics (AE), Bavarian Association of General Practitioners. The remaining authors declare no conflict of interest.

## ETHICS STATEMENT

The current study was approved by the Ethics Commission of the Medical Center, University of Heidelberg (S‐029/021). Written informed consent was obtained of every patient before inclusion.

## Supporting information

Supplementary information.

## Data Availability

The data will be available upon reasonable request.
