# Supplementary material for: Favourable clinical outcomes and low revision rate after M‐ACI in adolescents with immature cartilage compared to adult controls: Results at 10 years
Source: Knee Surg Sports Traumatol Arthrosc. 2024 Jul 15;33(1):167–76. doi: 10.1002/ksa.12359 (PMC11716355; doi:10.1002/ksa.12359)
Supplement: Supplementary file 1 — Supplementary information. [file KSA-33-167-s001.docx]

**Supplementary Table:**

Supplementary Table 1 Comparison of the IKDC, Tegner, VAS and EQ5D at the various FUs of the matched cohort

|  | **Adolescents** | **Adults** | **p-value** |
| --- | --- | --- | --- |
| **IKDC** |  |  |  |
| Baseline | 52.1 (33.6) | 46.3 (36.3) | (n.s.) |
| 12 Months | 56.0 (20.5) | 60.8 (25.0) | (n.s.) |
| 24 Months | 70.0 (14.7) | 68.1 (20.7) | (n.s.) |
| 96 Months | 79.4 (15.8) | 71.1 (13.9) | **0.0**2 |
| **Tegner** |  |  |  |
| Baseline | 2.8 (1.8) | 3.2 (2.0) | (n.s.) |
| 12 Months | 3.7 (1.8) | 4.0 (2.2) | (n.s.) |
| 24 Months | 4.5 (1.9) | 4.6 (2.1) | (n.s.) |
| 96 Months | 5.2 (2.3) | 4.5 (1.7) | (n.s.) |
| **VAS** |  |  |  |
| Baseline | 7.3 (2.2) | 7.2 (2.1) | (n.s.) |
| 12 Months | 3.6 (1.6) | 4.7 (1.3) | **0.0**4 |
| 24 Months | 3.1 (1.3) | 3.8 (1.6) | (n.s.) |
| 96 Months | 2.4 (1.7) | 3.0 (2.0) | (n.s.) |
| **EQ5D** |  |  |  |
| Baseline | 49.6 (26.1) | 56.7 (27.1) | (n.s.) |
| 12 Months | 64.5 (23.1) | 68.1 (23.3) | (n.s.) |
| 24 Months | 80.9 (14.9) | 77.1 (17.8) | (n.s.) |
| 96 Months | 89.5 (10.9) | 84.3 (10.8) | **0.01** |
